# Can Photoplethysmography Replace Arterial Blood Pressure in the Assessment of Blood Pressure?

**DOI:** 10.3390/jcm7100316

**Published:** 2018-09-30

**Authors:** Gloria Martínez, Newton Howard, Derek Abbott, Kenneth Lim, Rabab Ward, Mohamed Elgendi

**Affiliations:** 1 School of Electrical and Computer Engineering, University of British Columbia, Vancouver, BC V6T 1Z4, Canada; ketherelohim@gmail.com (G.M.); rababw@ece.ubc.ca (R.W.); 2 Center for Research and Advanced Studies (Cinvestav), Monterrey’s Unit, Apodaca N. L. 66600, México; 3 Nuffield Department of Surgical Sciences, University of Oxford, Oxford 450456, UK; newton.howard@nds.ox.ac.uk; 4 School of Electrical and Electronic Engineering, The University of Adelaide, Adelaide, SA 5005, Australia; derek.abbott@adelaide.edu.au; 5 Centre for Biomedical Engineering, The University of Adelaide, Adelaide, SA 5005, Australia; 6 Faculty of Medicine, University of British Columbia, Vancouver, BC V1Y 1T3, Canada; klim@cw.bc.ca; 7 BC Children’s & Women’s Hospital, Vancouver, BC V6H 3N1, Canada

**Keywords:** pulse morphology, pulse oximeter, blood pressure monitoring, pulse arrival time, global health, digital medicine, wearable devices

## Abstract

Arterial Blood Pressure (ABP) and photoplethysmography (PPG) are both useful techniques to monitor cardiovascular status. Though ABP monitoring is more widely employed, this procedure of signal acquisition whether done invasively or non-invasively may cause inconvenience and discomfort to the patients. PPG, however, is simple, noninvasive, and can be used for continuous measurement. This paper focuses on analyzing the similarities in time and frequency domains between ABP and PPG signals for normotensive, prehypertensive and hypertensive subjects and the feasibility of the classification of subjects considering the results of the analysis performed. From a database with 120 records of ABP and PPG, each 120 s in length, the records where separated into epochs taking into account 10 heartbeats, and the following statistical measures were performed: Correlation (*r*), Coherence (COH), Partial Coherence (pCOH), Partial Directed Coherence (PDC), Directed Transfer Function (DTF), Full Frequency Directed Transfer Function (ffDTF) and Direct Directed Transfer Function (dDTF). The correlation coefficient was r>0.9 on average for all groups, indicating a strong morphology similarity. For COH and pCOH, coherence (linear correlation in frequency domain) was found with significance (*p* < 0.01) in differentiating between normotensive and hypertensive subjects using PPG signals. For the dataset at hand, only two synchrony measures are able to convincingly distinguish hypertensive subjects from normotensive control subjects, i.e., ffDTF and dDTF. From PDC, DTF, ffDTF, and dDTF, a consistent, a strong significant causality from ABP→PPG was found. When all synchrony measures were combined, an 87.5% accuracy was achieved to detect hypertension using a Neural Network classifier, suggesting that PPG holds most informative features that exist in ABP.

## 1. Introduction

For decades, cardiovascular diseases (CVD) have been a major cause of mortality and morbidity around the world. Moreover, the number of patients suffering from CVD increases year by year. One of the major risk factors for the development of of CVD is chronic hypertension which is characterized by elevated baseline blood pressure over long periods of time. Chronic hypertension also increases the risk of cerebral vascular accidents and renal failure [1,2]. Therefore, the early detection of this hypertension is very important for its prevention and timely treatment. The blood pressure (BP) waveform is related to the transmission of arterial pulse waves, and is significant in the study of circulatory diseases.

There are two types of methodologies for BP monitoring: invasive and noninvasive. Depending on the clinical situation, either continuous or intermittent blood pressure monitoring is employed. For continuous monitoring, invasive (intravascular) central monitoring is employed which has potential risks to patients such as infection, site bleeding, arterial obstruction and distal limb ischemic damage and vascular damage. There are also clinical situations where invasive arterial monitoring may be difficult if not impossible to be performed safely. Therefore, a method of non-invasive continuous blood pressure monitoring may be valuable clinically.

There are many methods that can be used to noninvasively assess arterial blood pressure (ABP) waveforms; however, they are not user-friendly in practical applications. The photoplethysmogram (PPG) produces an optic signal related to arterial volumetric blood pulsations and has wide potential in clinical applications due to its simplicity and noninvasiveness [3,4]. Recently, it has been outlined as a potential alternative to cuff-based BP monitors [5]. Small sensor size and simple operation lead to PPG being widely applicable for the detection of cardiovascular and peripheral micro-circulation diseases [6,7]. Many important hemodynamic parameters can be evaluated from the PPG signal, such as heart rate, blood pressure, or pulse velocity [8,9,10]. Since the ABP and PPG signals have the same source of excitation (heart), it is logical to expect a similarity between the signals in both time and frequency; however, to our knowledge, there is no study that reports this.

Hypertension detection using cuff-based BP monitoring has been useful for the prevention of cardiovascular diseases for years. Some studies based on the measurement of PPG have reported suitable performance for the correct classification of patients when taking into account the cardiac variability [11,12,13]. However, these studies, and a recent study [14] by our group, considered only the characteristics in the time domain of the signal neglecting the frequency domain. Moreover, having the ABP as a reference to examine the suitability of the PPG signal is more accurate than correlating PPG signals with blood pressure reading collected using cuff-based BP monitors [15].

In the literature there are two approaches for determining BP using PPG signals: synchronicity-dependent approach (i.e., another biosignal is measured simultaneously with PPG signal) and asynchronicity-dependent approach (i.e., PPG signal alone) [14]. However, we are here trying to look deeply inside the PPG waveform and examine how similar and coherent it is with the ABP waveform, rather than focusing on extracting synchronicity-dependent and asynchronicity-dependent features and correlating them with blood pressure readings.

The main objective of this paper is to validate the potential use of PPG signals for BP evaluation. The proposed approach examines the similarity between PPG and ABP morphologies in time and frequency domains, as well as the changes that exist between normotensive, prehypertensive and hypertensive subjects. In order to establish the robustness of the PPG signal for BP monitoring with different types of patients and the feasibility of the classification of subjects considering the results of the analyses performed, which we describe below.

## 2. Methods

**Figure 1:**
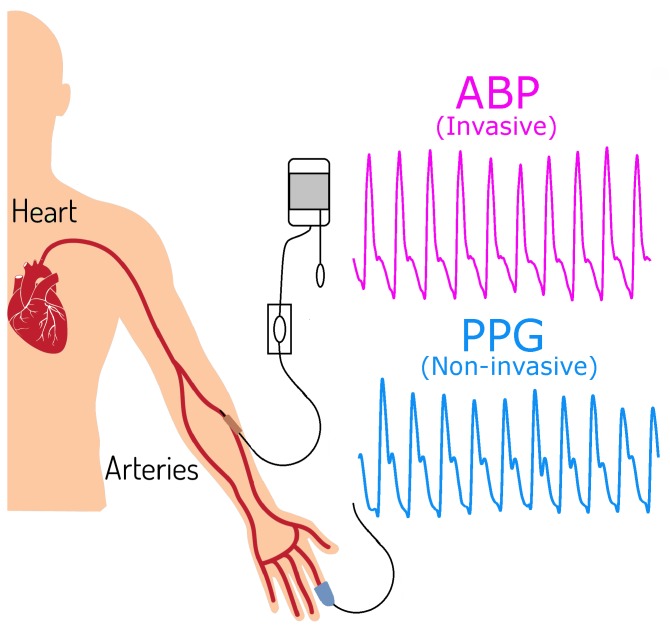
Simultaneously collected Arterial Blood Pressure (ABP) and Photoplethysmogram (PPG) signals. Note, ABP was measured invasively while PPG was measured non-invasively.

Our database, which is publicly available [16], called the MIMIC III (Multiparameter Intelligent Monitoring in Intensive Care) Database, consists of 120 subjects. Each subject was simultaneously measured by ABP and PPG, each signal 120 s in length and recorded with a sampling frequency (Fs) of 125 Hz (an example of the acquisition of the signals is shown in Figure 1). First, a separation of the subjects was performed according to the systolic BP (mmHg) categories of the American Heart Association (AHA). Note, some files that we used in our analysis are missing demographic information and therefore we could not study the PPG morphology changes with age.

For the purpose of this study, three groups were formed: Normal (less than 120 mmHg; n=43), prehypertensive (120–139 mmHg; n=40) and hypertensive (140 mmHg or higher; n=37). All signals were filtered with a bandpass filter in the 0.5–15 Hz range, normalized, and then separated into epochs that contain ten heartbeats. Subsequently, all epochs per subject were visually revised to discard those that had artifacts or in which signals were not well recorded. Finally, different analyses in time and frequency domain were performed with the filtered signals, where the signal ABP was denoted as X(t) and the signal PPG as Y(t).

### 2.1. Hypotheses

In the literature, there are two approaches for determining BP using PPG signals: synchronicity-dependent approach (i.e., another biosignal is measured simultaneously with PPG signal) and asynchronicity-dependent approach (i.e., PPG signal alone) [14]. However, we are here trying to look deeply inside the PPG waveform and examine how similar and coherent it is with the ABP waveform, rather than focusing on extracting synchronicity-dependent and asynchronicity-dependent features and correlating them with blood pressure readings. We have multiple hypotheses to address the questions raised in the title of the paper:

- H1: If the PPG amplitude (Linear time domain analysis) is correlated with the ABP amplitude, then the PPG amplitude can replace ABP for measuring BP.
- H2: If the PPG morphology (Linear time domain analysis) is correlated with the ABP morphology, then the PPG waveform morphology holds valuable information that can be used for evaluating BP.
- H3: If the PPG waveform and the ABP have mutual information and coherence (Nonlinear dependency analysis), then the PPG waveform morphology holds valuable information that can be used for evaluating BP.

Note that H1, and H3 are out-phase analyses (no shifting step applied to the signals) while H2 is an in-phase analysis (signals are shifted and aligned). The in-phase and out-phase analyses are described below.

### 2.2. In-Phase Analysis

The in-phase analyses were carried out per epoch by aligning PPG and ABP waveforms. Pearson’s correlation coefficient is used to determine how similar PPG and ABP are in terms of morphology. The correlation coefficient *r* can be calculated by Equation (1). The value of *r* ranges from −1 to +1, where the value close to +1 or −1 means that signals have strong positive or negative similarity, respectively; otherwise, the value is close to zero. The correlation coefficient is calculated as follows:

(1) $$\begin{array}{c} r=\frac{n\sum XY-\sum X\sum Y}{\sqrt{n\sum X^{2}-{\left(\sum X\right)}^{2}}-\sqrt{n\sum Y^{2}-{\left(\sum Y\right)}^{2}}}. \end{array}$$

The results of the epochs were averaged per subject.

### 2.3. Out-Phase Analysis

The out-phase analyses were applied to the ABP and PPG signals without any adjustments in the time domain. Six analyses were performed: Coherence (COH), Partial Coherence (pCOH), Partial Directed Coherence (PDC), Directed Transfer Function (DTF), Full Frequency Directed Transfer Function (ffDTF) and Direct Directed Transfer Function (dDTF). These metrics aim to determine coupling, direct coupling, direct causality, directed connectivity, and direct and indirect connection as functions of frequency [17]. Their values range from 0 to 1, where 1 means total connectivity or causality, and zero means non-connectivity.

For each epoch, an autoregressive (AR) model was estimated [18], where the dependence of the signals was given by

(2) $$\begin{array}{c} \tilde{X}\left(t\right)=\sum_{k=1}^{p}a_{11}\left(k\right)\tilde{X}\left(t-k\right)+\sum_{k=1}^{p}a_{12}\left(k\right)\tilde{Y}\left(t-k\right)+u_{1}\left(t\right) \end{array}$$

(3) $$\begin{array}{c} \tilde{Y}\left(t\right)=\sum_{k=1}^{p}a_{21}\left(k\right)\tilde{Y}\left(t-k\right)+\sum_{k=1}^{p}a_{22}\left(k\right)\tilde{X}\left(t-k\right)+u_{2}\left(t\right) \end{array}$$

where $a_{ij}\left(k\right)$ is the model coefficients, $u_{1}\left(t\right)$ is the prediction error when $\tilde{X}\left(t\right)$ is predicted from its own past and the past of $\tilde{Y}\left(t\right)$, and $u_{2}\left(t\right)$ is similar to $u_{1}\left(t\right)$. The optimal model order *p* was calculated with the Bayesian criterion and was fixed the same for all the epochs per group. The AR model can be expressed in matrix form as $\xi \left(t\right)={\left[\tilde{X}\left(t\right),\tilde{Y}\left(t\right)\right]}^{T}$, $\eta ={\left[u_{1}\left(t\right),u_{2}\left(t\right)\right]}^{T}$ and $A\left(k\right)=-(a_{ij}\left(k\right),i,j=1,2)$ as

(4) $$\begin{array}{c} \xi \left(t\right)=-\sum_{k=1}^{p}A\left(k\right)\xi \left(t-k\right)+\eta \left(t\right). \end{array}$$

With the identity matrix A(0)=I, Equation (4) can be rewritten as $\sum_{k=0}^{p}A\left(k\right)\xi \left(t-k\right)=\eta \left(t\right)$. The spectral relationship of this equation can be written as A(f)ξ(f)=η(f), in which $\xi \left(f\right)=A^{-1}\left(f\right)\eta \left(f\right)=H\left(f\right)\eta \left(f\right)$ and $A\left(f\right)=\sum_{k=0}^{p}A\left(k\right)e^{\left(-ik2\pi f\right)}$.

The power spectral matrix of signals is given by

(5) $$\begin{array}{c} S\left(f\right)=\xi^{*}\left(f\right)\xi \left(f\right)=H\left(f\right)\eta \left(f\right)\eta^{*}\left(f\right)H^{*}\left(f\right)=H\left(f\right)\sum H^{*}\left(f\right), \end{array}$$

where * stands for conjugate transpose, $\Sigma =(\Sigma_{ij},i,j=1,2)$ is the covariance matrix of η(t), and the coherence is computed by

(6) $$\begin{array}{c} {\mathrm{COH}}_{XY}\left(f\right)=\frac{\left|S_{XY}\left(f\right)\right|}{\sqrt{S_{XX}\left(f\right)S_{YY}\left(f\right)}}, \end{array}$$

defining $P\left(f\right)=S{\left(f\right)}^{-1}$, the partial coherence is calculated as

(7) $$\begin{array}{c} {\mathrm{pCOH}}_{XY}\left(f\right)=\frac{\left|P_{XY}\left(f\right)\right|}{\sqrt{P_{XX}\left(f\right)S_{YY}\left(f\right)}}. \end{array}$$

The PDC is defined by [19] in the following form

(8) $$\begin{array}{c} {\mathrm{PDC}}_{ij}\left(f\right)=\frac{A_{ij}\left(f\right)}{\sqrt{a_{j}^{*}\left(f\right)a_{j}\left(f\right)}}, \end{array}$$

where $A_{ij}\left(f\right)$ is an element of A(f) and $a_{j}\left(f\right)$ is the *j*th column of A(f) and the asterisk denotes the transpose and complex conjugate operation.

The DTF was introduced in [20] and is determined as

(9) $$\begin{array}{c} {\mathrm{DTF}}_{j\to i}\left(f\right)=\frac{\left|H_{ij}\left(f\right)\right|}{\sqrt{\sum_{m=1}^{k}{\left|H_{im}\left(f\right)\right|}^{2}}}. \end{array}$$

The ffDTF is a modification of the DTF concerning the normalization of the function in such a way as to make the denominator independent of frequency and is given by

(10) $$\begin{array}{c} {\mathrm{ffDTF}}_{ij}\left(f\right)=\frac{\left|H_{ij}\left(f\right)\right|}{\sqrt{\sum_{f}\sum_{m=1}^{k}{\left|H_{im}\left(f\right)\right|}^{2}}}, \end{array}$$

while the dDTF is defined as a multiplication of ffDTF by pCOH and shows direct propagation from channels *j* to *i*. It is calculated as

(11) $$\begin{array}{c} {\mathrm{dDTF}}_{ij}\left(f\right)={\mathrm{ffDTF}}_{ij}\left(f\right)\;{\mathrm{pCOH}}_{ij}\left(f\right). \end{array}$$

These analyses were calculated by taking into account the maximum value in the frequency range of 1–10 Hz per epoch, and the results of epochs per subject were averaged.

### 2.4. Statistical Analysis

Two statistical nonparametric tests were performed with the results obtained from the analyses in the time and frequency domains to find any differences between the groups of normotensive (NT), prehypertensive (PHT), and hypertensive (HT): Wilcoxon Rank Sum test (α=0.1) and Kruskall–Wallis (K–W) test were used for the results of comparing two groups and three groups, respectively.

### 2.5. Classification Analysis

A classification analysis with two categories, NT and HT, was performed with the results of the analysis in the frequency domain. Twelve classifiers were evaluated with the cross validation technique (leave-one-out) with five Granger’s measures defined as follows: COH, PDC, DTF, ffDTF, dDTF, and all five measures combined as one feature set.

## 3. Results and Discussion

**Figure 2:**
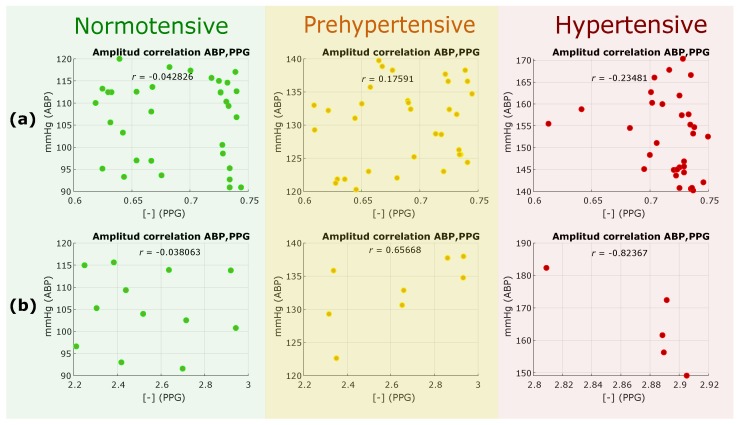
Results of in-phase analysis: (**a**) Analysis results for the group with an amplitude range of 0.6–0.75 of the PPG signal; and (**b**) analysis results for the group with an amplitude range of 2.2–3 of the PPG signal. The groups are derived from the different gains of the equipment with which the signals were registered. *r* refers to the morphology correlation between photoplethysogram (PPG) and arterial blood pressure (ABP) waveforms.

We tested the H1 hypothesis, which is the out-phase analysis, to see if the PPG amplitude is correlated with the ABP amplitude. If correlated, then the PPG amplitude can replace ABP for measuring BP. We extracted the amplitude of each PPG waveform and the amplitude of its corresponding ABP waveform, and then scattered these amplitudes to examine correlation. As the MIMIC database was created and collected from different sources and used different clocks [21], we split the PPG data into two categories of the same electronic gain, 0.6–0.75 and 2.2–3, as shown in Figure 2a,b, respectively. The overall results, as shown in Figure 2, show that the amplitudes of PPG waveforms and their corresponding ABP waveforms are not correlated. Therefore, the outcome of this analysis rejected the H1 hypothesis, and PPG amplitudes cannot replace ABP amplitudes.

**Figure 3:**
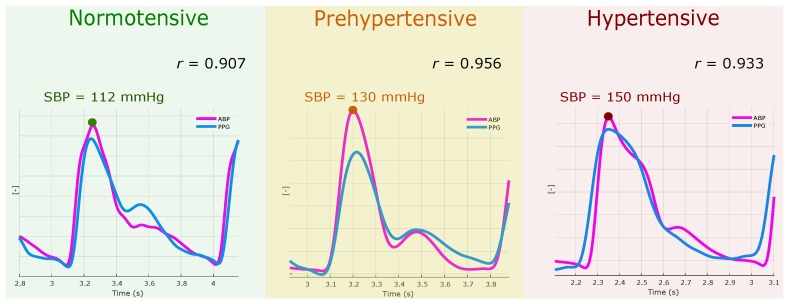
Examples of in-phase analysis. Note, *r* refers to the morphology correlation between photoplethysogram (PPG) and arterial blood pressure (ABP) waveforms.

The rejection of the H1 hypothesis confirms and validates testing the H2 and H3 hypothesis. The results of the H2 hypothesis, which is in-phase analyses, to determine the time-domain correlation between the ABP and PPG morphologies when aligned, are shown in Figure 3. Note that the peaks of the systolic waves in both ABP and PPG signals were adjusted to the same position to test the morphology correlation. As shown in Figure 3, the correlation coefficients were significant in hypertensive, normotensive and prehypertensive cases.

On average, the correlation coefficient r>0.9 was obtained (see Table 1), and therefore this result suggest acceptance of H2 hypothesis. This implies that both signals had a high similarity in their morphology; however, as the ABP increased, different phases between the signals appeared. This could be due to the decrease in the time it took for the blood to travel to the extremities with a high pressure, thus advancing the appearance of the PPG signal. A good correlation in time domain and frequency domain features of radial PPG and ABP was reported [22]. However, the change of this correlation has not been studied according to the BP increases. From the obtained results, it can be inferred that the use of the PPG signal is robust for monitoring BP and that there is a significant change between N and H groups due to the reduction of the delay of the PPG signal.

Table 1 is valuable for researchers who are interested in selecting recordings from the MIMIC database that contain PPG waveforms correlated with ABP waveforms. At least, based on objective measures such as correlation, inclusion and exclusion criteria, recording selection can be carried out regardless of the study aim. For example, researchers can include only recordings that have r>0.9 in their analysis, rather than including recording based on subjective methods such as visual inspection.

The results of the H3 hypothesis, which is an out-phase analyses for time and frequency domains, can be seen in Table 2. The *p*-value of the Wilcoxon test corresponds to where the difference was found in comparing NT vs. PHT and NT vs. HT, while Kruskall–Walllis test for comparing NT vs. PHT vs. HT. As can be seen in Table 2, the first tested measure was the correlation coefficient; however, we are expecting that there will be no correlation between ABP and PPG morphologies as it is out-phase analysis. Interestingly, COH and pCOH scored the exact *p*-values for all comparisons, with a significant difference (p=0.0069) in comparing NT vs. HT groups, this result suggest acceptance of the H3 hypothesis.

The results, as shown in Table 2, suggest that, taking the median of these results into account, when ABP increases, the coupling of the signals increases as well. Studies in the frequency domain of these signals have focused mainly on the spectrum they handle and their relationship with the heart rate variability (HRV) [23]. The fact that the coupling of the signals improves as the ABP increases might be because the phase between the signals decreases, and it should be noted that N and H groups can be differentiated. For the rest of the analyses (PDC, DTF, ffDTF, and dDTF), two results were derived: PPG→ABP and ABP→PPG causalities.

**Figure 4:**
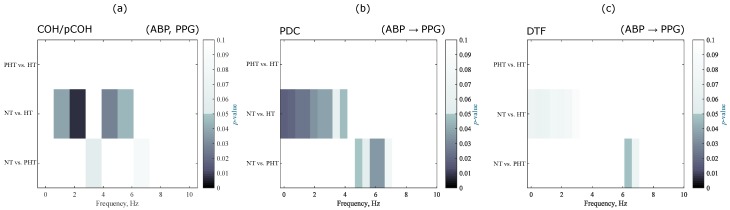
Frequency significance in differentiation blood pressure groups: (**a**) COH and pCOH (**b**) PDC direction ABP→PPG; and (**c**) DTF direction ABP→PPG. Abbreviations: NT, normotensive; PHT, Prehypertensive; HT, Hypertensive; COH, Coherence; pCOH, Partial coherence; PDC, Partial directed coherence; DTF, Directed transfer function; dDTF, Direct directed transfer function; ffDTF, Full frequency directed transfer function.

Moreover, we evaluated the range of frequencies in which the groups can be differentiated for COH, pCOH, PDC and DTF. The latter were analyzed only in the direction ABP→PPG, as is shown in Figure 4.

These types of metrics are mainly used to determine the causality, connectivity, and synchronicity between brain signals [24,25] and changes in the brain during a specific activity [26]. The use of some of these has been reported in cardiovascular signals, where the principal purpose was to study the characteristics of the pulse transit time (PTT), and the ABP and BP waveforms in normotensive subjects [27]. The results of the analyses in the frequency domain were consistent, indicating that the causality of connectivity between the signals goes ABP→PPG, which is in agreement physiologically with the measurement sites of both signals.

The different metrics of Granger’s causality allow us to see the directionality of the relationship of the signals, and not only the level of this relationship. The results obtained were expected due to the physiological measurement location of the ABP and PPG sensors. The ABP→PPG direction infers that the ABP signal is first and it influences the PPG signal. Results mathematically show that changes in blood pressure cause changes in blood volume, not the other way around.

**Figure 5:**
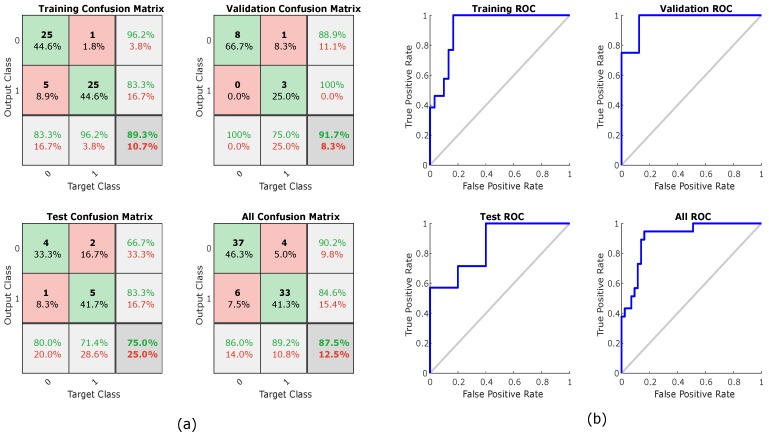
Neural networks training, testing, and validation using all causality measures: (**a**) confusion plot; and (**b**) receiver operating characteristic (ROC) curve.

The results of the classification analysis are shown in Table 3. The highest classification percentage (87.5%) was obtained with a two-layer feed forward Neural Network classifier that uses all five Granger’s measures as a feature set. In Figure 5 can be see the confusion plots and the ROC curve plots of the best classification.

**Figure 6:**
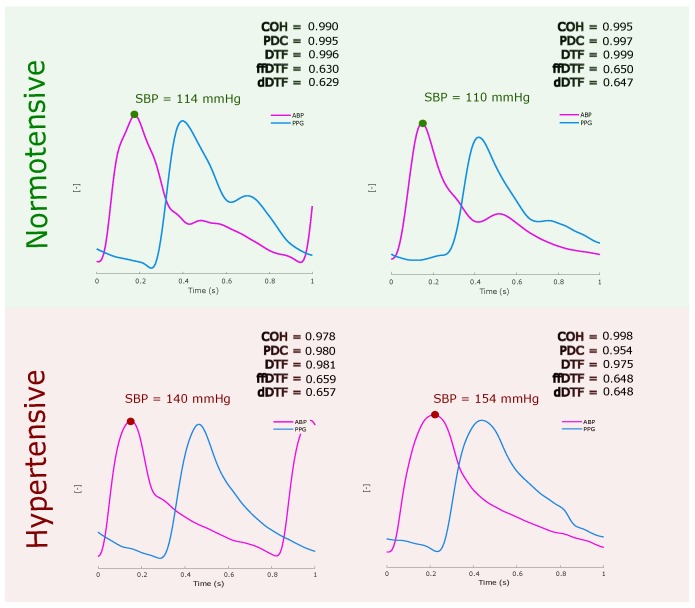
Examples of different causality measures for normotensive and hypertensive cases. Abbreviations: COH, Coherence; pCOH, Partial coherence; PDC, Partial directed coherence; DTF, Directed transfer function; dDTF, Direct directed transfer function; ffDTF, Full frequency directed transfer function.

The results obtained in the frequency domain had a clear incremental trend parallel to the BP, and this implies that the two groups (NT and HT) can be differentiated even when the similarity-causality between the signals is high in all groups. Figure 6 shows an example of this, where a peak of the ABP signal (pink color) and its corresponding PPG (light blue) are shown for normotensive and hypertensive subjects and their corresponding results of each of the calculated measures.

Based on the out-phase analysis shown Figure 6, the ABP and PPG morphologies in the frequency domain are coherent. Some measures are more sensitive than others; however, examining all measures confirms the coherence and similarity between the ABP and PPG signals. Note that the results show a great morphological similarity between the signals of ABP and PPG regardless if age is confounding or not.

During the out-phase analysis, it was assumed that, since the ABP and PPG signals were simultaneously collected (i.e., all signals were collected at the same time with no delay), the signals were also synchronized. Many papers published results using the MIMIC database and assumed that all signals were collected at the same time and synchronized [28,29,30,31,32]. However, even if a delay (or error in the data matching and alignment) occurred in some recordings, as mentioned in [21], the analysis is still important as it sheds light on the overall synchronicity trend over all recordings.

Several algorithms can be used to calculate the BP based on the PPG signal along with other biosignals [33,34]. While it is true that new technologies and methodologies are still being developed to make collection of multiple biosignals more portable and precise, there is still a lot of research and testing needed [35,36]. Our study focused on providing evidence that the PPG morphology is similar to (and as informative as) the ABP morphology in both the temporal and frequency domain, and thus the PPG could be used instead of ABP. Further research needs to be done on how PPG can be used in clinical and remote settings.

## 4. Limitation of Study and Future Work

Our main goal here was to examine the similarities between PPG and ABP waveforms. We did not focus on optimizing the classifier or evaluating BP using PPG signals. One of our next steps is to estimate blood pressure from PPG waveform based on the findings of this paper.

The main limitations of the files used in this paper were the lack of demographic and clinical data, as well as a lack of more detailed information on the equipment used and the relative calibrations for the records. We did the analysis without looking into any confounding factors. On the other hand, although many papers have published results using the MIMIC database and it is assumed that all the signals were collected at the same time and synchronized, this is not the case, which made it impossible to perform further frequency analyses. As future work, we would like to apply the methodologies proposed in other databases and even in data acquired by us to have precise control of the gains of the sensors, and propose a metric for the calculation of the SBP and DBP from the PPG signal that goes according to our analysis.

## 5. Conclusions

From the results of the analyses carried out, it can be concluded that there is a similarity between the PPG and ABP morphologies. This is reflected in the correlation coefficient calculated in the time-domain in-phase analysis. From the frequency domain analysis, it can be concluded that, as the BP increases, the synchronicity of the signals also increases, in addition to the higher causality is given ABP → PPG, which already mentioned above has congruence for the physiological relevance of the registration of the signals. Finally, the results of the classification analysis with the combination of the most two informative frequency measures suggest that PPG signal can be used to determine if a patient is normotensive or hypertensive similar to the ABP signal. The main conclusion of this work is that BP monitoring through PPG is a very promising for evaluating BP and it is a potentially feasible replacement for invasive ABP.

## Figures and Tables

**Table 1:** In-phase correlation between photoplthysmogram (PPG) and arterial blood pressure (ABP) signals.

Normotensive		Prehypertensive		Hypertensive	
Subject ID	*r*	Subject ID	*r*	Subject ID	*r*
’11727_2’	0.9073	’12531_2’	0.8385	’10464_2’	0.9327
’12174_2’	0.9142	’13600_2’	0.916	’11187_2’	0.906
’17848_2’	0.9676	’15218_2’	0.9797	’1501_2’	0.9023
’19700_2’	0.951	’15716_2’	0.9286	’15902_2’	0.9696
’2104_2’	0.9459	’16129_2’	0.9557	’18642_2’	0.9448
’2513_2’	0.9201	’18970_2’	0.9025	’19578_2’	0.9492
’27436_2’	0.9806	’21730_2’	0.7777	’20726_2’	0.9109
’27648_2’	0.9689	’26897_2’	0.9317	’22335_2’	0.947
’27833_2’	0.9354	’27241_2’	0.9359	’23201_2’	0.9558
’27887_2’	0.802	’27337_2’	0.9384	’2458_2’	0.9744
’28077_2’	0.9362	’27434_2’	0.8639	’27446_2’	0.7908
’28187_2’	0.9633	’27845_2’	0.9413	’28499_2’	0.9787
’28813_2’	0.9802	’28758_2’	0.8939	’28510_2’	0.921
’28910_2’	0.9699	’28882_2’	0.9833	’28775_2’	0.8816
’29102_2’	0.9178	’44088_2’	0.8657	’29127_2’	0.978
’29120_2’	0.9382	’44104_2’	0.9413	’44118_2’	0.9415
’3039_2’	0.9481	’44201_2’	0.9773	’44171_2’	0.9377
’44223_2’	0.9533	’44233_2’	0.9592	’44173_2’	0.969
’44409_2’	0.9441	’44458_2’	0.8442	’44347_2’	0.8966
’44422_2’	0.9672	’44496_2’	0.9571	’44572_2’	0.93
’44432_2’	0.956	’44590_2’	0.9745	’44615_2’	0.8795
’44526_2’	0.8325	’44623_2’	0.945	’44616_2’	0.9505
’44598_2’	0.9419	’44640_2’	0.9663	’44626_2’	0.9526
’44601_2’	0.9499	’44647_2’	0.9771	’44704_2’	0.9689
’44629_2’	0.9757	’44902_2’	0.9434	’44839_2’	0.9742
’44671_2’	0.9612	’45181_2’	0.8832	’44981_2’	0.9583
’44758_2’	0.9754	’45384_2’	0.9142	’45098_2’	0.9663
’44763_2’	0.9261	’45426_2’	0.8659	’45140_2’	0.9561
’44783_2’	0.9333	’45533_2’	0.9798	’45212_2’	0.9223
’44810_2’	0.8872	’45572_2’	0.8721	’45227_2’	0.8929
’45049_2’	0.9529	’45636_2’	0.9239	’45550_2’	0.9355
’45186_2’	0.9373	’45641_2’	0.9278	’45627_2’	0.902
’45311_2’	0.9615	’46138_2’	0.9157	’46216_2’	0.8924
’45343_2’	0.9688	’46297_2’	0.8783	’46303_2’	0.9499
’45353_2’	0.8794	’46416_2’	0.7969	’801_2’	0.9454
’45487_2’	0.9774	’4679_2’	0.849	’8141_2’	0.9484
’45556_2’	0.9586	’6581_2’	0.9256	’8318_2’	0.871
’45645_2’	0.9703	’6692_2’	0.9332		
’46122_2’	0.981	’7614_2’	0.908		
’46230_2’	0.9661	’9124_2’	0.9677		
’46424_2’	0.9086				
’5937_2’	0.9206				
’946_2’	0.9474				
**Average**	**0.9414**		**0.917**		**0.932**

**Table 2:** Out-phase statistical separability results. Abbreviations: NT, normotensive; PHT, Prehypertensive; HT, Hypertensive; *r*, Pearson’s correlation coefficient; COH, Coherence; pCOH, Partial coherence; PDC, Partial directed coherence; DTF, Directed transfer function; dDTF, Direct directed transfer function; ffDTF, Full frequency directed transfer function; K-W test, Kruskal–Wallis test.

**Time Domain**	**NT vs. PHT**	**NT vs. HT**	**PHT vs. HT**	***p*-Value (K–W Test)** **NT vs. PHT vs. HT**
	**p-value (Wilcoxon Test)**			
*r*	0.4466	0.4552	0.9600	0.6726
**Frequency domain**	**p-value (Wilcoxon Test)**			**p-value (K–W test)** **NT vs. PHT vs. HT**
COH & pCOH	0.2355	0.0069	0.1630	0.0281
**PPG → ABP**	**p-value (Wilcoxon Test)**			**p-value (K–W test)** **NT vs. PHT vs. HT**
PDC	0.6258	0.1861	0.4536	0.4248
DTF	0.5324	0.2061	0.4475	0.4219
ffDTF	0.2527	0.2928	0.9715	0.4352
dDTF	0.2681	0.2973	0.9552	0.4506
**ABP → PPG**	**p-value (Wilcoxon Test)**			**p-value (K–W test)** **NT vs. PHT vs. HT**
PDC	0.0264	0.0102	0.7250	0.0188
DTF	0.1788	0.0479	0.5995	0.1304
ffDTF	0.0762	0.0022	0.1128	0.0064
dDTF	0.0718	0.0020	0.1249	0.0061

**Table 3:** Classification accuracy results. Abbreviations: COH, Coherence; pCOH, Partial coherence; PDC, Partial directed coherence; DTF, Directed transfer function; dDTF, Direct directed transfer function; ffDTF, Full frequency directed transfer function.

#	Classifier	COH	PDC	DTF	ffDTF	dDTF	All Features
1.1	Linear	66.3%	51.3%	53.8%	65.0%	62.5%	70.0%
1.2	Diaglinear	66.3%	51.3%	53.8%	65.0%	62.5%	70.0%
1.3	Quadratic	60.0%	52.5%	50.0%	62.5%	62.5%	58.8%
1.4	Diagquadratic	60.0%	52.5%	50.0%	62.5%	62.5%	65.0%
1.5	Mahalanobis	70.0%	52.5%	48.8%	61.3%	61.3%	65.0%
1.6	SVM	66.3%	55.0%	53.7%	56.2%	56.2%	67.5%
1.7	KNN	63.8%	45.0%	56.2%	47.5%	47.5%	71.3%
1.8	Tree	71.3%	57.5%	**65.0%**	52.5%	57.5%	60.0%
1.9	Naive Bayes	62.5%	52.5%	50.0%	62.5%	62.5%	65.0%
1.10	Ecoc	53.7%	53.7%	53.7%	53.7%	53.7%	60.0%
1.11	Esemble	63.8%	51.2%	50.0%	53.7%	56.2%	62.5%
1.12	Two-layer feed forward Neural Network	**77.5%**	**66.3%**	63.7%	**78.8%**	**67.5%**	**87.5%**
